# Comparison of the Prevalence of Psychiatric Disorders in Performance-Enhancing Drug Users and Nonuser Bodybuilders 

**Published:** 2017-10

**Authors:** Afshin Ostovar, Mohammad Javad Haerinejad, Samad Akbarzadeh, Mojtaba Keshavarz

**Affiliations:** 1 The Persian Gulf Tropical Medicine Research Center, Bushehr University of Medical Sciences, Bushehr, Iran.; 2 Osteoporosis Research Center, Endocrinology and Metabolism Research Institute, Tehran University of Medical Sciences, Tehran, Iran.; 3 Department of Biochemistry, Bushehr University of Medical Sciences, Bushehr, Iran.; 4 Shiraz Neuroscience Research Center, Shiraz University of Medical Sciences, Shiraz, Iran.; 5 Department of Pharmacology, Bushehr University of Medical Sciences, Bushehr, Iran.

**Keywords:** *Bodybuilder*, *Mental Disorders*, *Performance-Enhancing Drugs*

## Abstract

**Objective: **The present study aimed at comparing the prevalence of major psychiatric disorders including major depressive disorder, bipolar disorder, schizophrenia, and generalized anxiety disorder between performance-enhancing drug users and nonuser bodybuilders. Moreover, the prevalence of major psychiatric disorders in bodybuilders was also reported.

**Method:** In this study, 453 athletes were recruited from Bushehr bodybuilding gyms from February to May 2015. A structured questionnaire was used to collect the participants’ information, including demographic characteristics, sports’ status and performance-enhancing drug use. According to the condition of performance-enhancing drug use, the participants were divided into current users, non-current users, and nonusers. The psychiatric status of the participants was evaluated using DSM-IV diagnostic criteria for major depressive disorder, bipolar disorder, generalized anxiety disorder, and schizophrenia. We also asked about the acute psychotic disturbances after using performance-enhancing drugs, alcohol use, and history of aggressive behavior in bodybuilders. Data were analyzed using one-way analysis of variance and chi-square tests.

**Results: **Prevalence of major depressive disorder, bipolar disorder, schizophrenia, generalized anxiety disorder, and the overall prevalence of psychiatric disorders in the bodybuilders was 19.7%, 3.8%, 1.5%, 16.6%, and 26.7%, respectively. After using performance-enhancing drugs, 33% of the bodybuilders had experienced acute psychological disturbances. There were no significant differences between current, non-current, and nonuser bodybuilding athletes in the measured psychiatric disorders.

**Conclusion: **Prevalence of psychiatric disorders was not significantly different in performance-enhancing drug users and nonusers. Thus, it can be concluded that performance-enhancing drugs do not increase the risk of psychiatric disorders in bodybuilders.

The advancing rate of performance-enhancing drugs (PED) abuse in many countries around the world poses the public health to serious problems and this issue is not well appreciated by the medical community or official authorities (1, 2). PED abuse, particularly anabolic steroids (AASs), can produce adverse effects on different body systems including endocrine system, reproductive system, liver, skin, cardiovascular system, and central nervous system (CNS) (3, 4). In spite of the numerous studies that assessed the somatic effects of AASs, only a few examined the psychiatric adverse effects of these agents (5).

Furthermore, there is limited information about the mental effects of the concurrent use of other agents such as stimulants in combination with AASs.

Taken together, psychiatric side effects of PEDs, particularly AASs, in the bodybuilders is an important challenging issue. Moreover, most studies did not examine the psychiatric effects of the concurrent use of other drugs such as stimulants in addition to the AASs in bodybuilders (13). The present study aimed at comparing the prevalence of major psychiatric disorders including major depressive disorder, bipolar disorder, schizophrenia, and generalized anxiety disorder between male PED users and nonuser bodybuilders. We also reported the prevalence of psychiatric disorders in male bodybuilders.

## Materials and Methods

*Participants and Study Design*

Sample size (N = 474) was calculated via power and sample size calculator software (Version 3.1.2) by considering the power of 0.80, α of 0.05, P0 of 0.10, and P1 of 0.05. We randomly selected 11 bodybuilding clubs from Bushehr port and invited all athletes, who were present at the club. Males with regular training routines (minimum of 1 year and 4 hours/week) were eligible to participate in the study. Those with the history of drug abuse, psychoactive drug use, and chronic disease were excluded from the study. In our study, 453 male bodybuilders participated from February to May 2015. 

All parts of the survey were in accordance with the Declaration of Helsinki and approved by the research ethics committee of Bushehr University of Medical Sciences. We presented all the details of the study and ensured the participants that all parts of the survey would remain confidential and would only be used collectively for the research purposes. After introducing all aspects of the survey, the participants were asked to sign written consent forms. 

*Data Collection*

Before the initiation of the study, the validity and reliability of the study questionnaire were demonstrated by Waltz-Bausell (CVI = 0.83) and Cronbach (alpha = 0.744) methods on a group of bodybuilders, who went to the university gym. The questionnaires were completed by a trained physician who had experience in psychiatric evaluation. The questionnaire was mostly obtained from Ip et al. (14) investigation and evaluated several characteristics of male bodybuilders in 3 separate parts. In the first part, we recorded the demographic characteristics such as age, height, and weight. In the second part, we asked about sports’ status (recreational or professional), sports history, exercise schedule, and PEDs use (the age of first use, the type of PED, the period of use, and the length of PED cycles in each year). To confirm PED use, we assessed the follicular stimulating hormone (FSH) and luteinizing hormone (LH) by the chemiluminescence method using commercial kits (Roche, USA). Then, the athletes were classified according to the status of PEDs use. Athletes with lower than normal level of gonadotropins (lower than 1.0 MIU/ml for FSH and lower than 0.7 MIU/ml for LH) were categorized as current users. Athletes with a history of PED use within 24 months and a normal level of FSH and LH were considered as non-current users, and those with normal level of gonadotropins and no history of PED use were considered as nonusers. Reasonably, this classification is only applicable for the AASs. In the third part, we asked about the family history and personal history of psychiatric disorders. Then, we evaluated the psychiatric status of the participants using DSM-IV diagnostic criteria for MDD, BD, GAD, and schizophrenia. Moreover, we asked about alcohol use, history of aggressive behavior, and acute psychotic disturbances after using PEDs (euphoria, irritability, mood swings, violent feelings, and hostility).

*Statistical Analysis*

Descriptive data were reported as mean ± SEM for the continuous data and number and percentage of the participants for the categorical data. We used Student’s t test and one-way analysis of variance (ANOVA) test for the continuous data, and chi-square test for the categorical data. Spearman test was used to assess the correlation between the variables. Statistical significance was set at 0.05 probability level. We performed all statistical analyses using SPSS software for Windows, Version 23.

## Results

*Demographic and Sports Status of the Bodybuilder*

In the present study, 4.2% (n = 19) of the participants were current users, 47.5% (n = 215) non-current users, and 48.3% (n=219) nonuser athletes. Moreover, 79.4% of the participants reported AAS use, 49.6% stimulants, and 7.7% peptide hormones. The age, weight, and BMI of the 3 study groups were significantly different (Table 1). Post-hoc analysis showed that the age of current users (p=0.014) and non-users (p=0.010) were higher than the non-users. The BMI and weight of the non-current users were higher than the non-users (p=0.000). Moreover, the mean sports history (year) of current and noncurrent users were significantly higher than the nonuser athletes (Table 1). In addition, the exercise schedules of the 3 studied groups were significantly different (Table 1). Post-hoc analysis showed that the exercise schedule of current (p=0.008) and non-current (p=0.008) users were more rigorous than the non-users. Table 1 also demonstrates sports status and characteristics of PED use in bodybuilders.

The prevalence of MDD, BD, schizophrenia, GAD, and the overall prevalence of psychiatric disorders in the bodybuilders was 19.7%, 3.8%, 1.5%, 16.6%, and 26.7%, respectively (table2). Furthermore, 8.8% of all participants had a family history of psychiatric disorders.

**Table1:** The Demographic Variables, Sports Status, and Characteristics of Performance-Enhancing Drug Use in the Bodybuilders

	**Current users ** **(Mean ± SEM)**	**Non-current users ** **(Mean±SEM)**	**Nonusers ** **(Mean±SEM)**	**F(df)**	**p**
Age	30.42±1.46	27.82±0.42	25.95±0.48	7.02(2)	0.00
Weight	82.32±2.55	83.14±0.89	77.68±0.78	11.06(2)	0.00
BMI†	26.74±0.81	26.32±0.26	24.84±0.23	10.15(2)	0.00
Sport history (year)	8.37±1.25	6.77±0.37	4.74±0.30	11.45(2)	0.00
Exercise routines (hour/week)	8.74±0.86	8.80±0.27	7.64±0.20	6.10(2)	0.00
Age of first use (year)	24.84±1.34	23.34±0.38	-	1.27(1)	0.26
Length of PED‡ use cycles (day/year)	74.74±9.14	59.42±2.58	-	2.86(1)	0.09
History of PED use (year)	3.26±0.58	3.23±0.28	-	0.00(1)	0.98

† BMI: body mass index,

‡ PED: performance-enhancing drug

**Table2:** Prevalence of Psychiatric Disorders in the Male Bodybuilders

	**Current PED users ** **(%)**	**Non-current PED ** **users (%)**	**Nonusers ** **(%)**	**Total ** **(%)**	χ**2(df)**	**P-value**
MDD	15.8	22.3	17.3	16.6	0.88(2)	0.64
BD	0	4.7	3.2	3.8	0.92(2)	0.63
Schizophrenia	0	1.9	1.4	1.5	0.36(1)	1.00
GAD	15.8	19.5	13.7	16.6	0.16(1)	1.00
OPP	26.3	30.7	22.8	26.7	0.16(1)	0.80

The data were analyzed using Chi-square test. The p-value less than 0.05 was considered to be significant.

**Table3:** Prevalence of Violence and Alcohol Use in the Male Bodybuilders

	**Current PED users (%)**	**Non-current PED users (%)**	**Nonusers (%)**	**Total (%)**
Alcohol use	52.6*	44.2*	26.5	36
Violence	36.8*	15.4*	5.5	11.7

* p value of <0.05 compared to the nonusers

**Table4:** The comorbidity of Violence and Alcohol Use with Psychiatric Disorders in the Male Bodybuilders

		**Violence ** **(%)**	χ^2^	**p-value**	**Alcohol use (%)**	χ^2^	**p-value**
Major depressive disorder	Healthy	8.2	21.44	0.000	31.9	13.61	0.000
		25.8			52.8		
	Depressed						
Bipolar disorder (BD)	Healthy	11	5.36	0.037	35.6	0.94	0.440
		29.4			47.1		
	Patient						
Schizophrenia	Healthy	11.2	6.68	0.038	35.4	3.88	0.104
		42.9			71.4		
	Patient						
Generalized anxiety disorder	Healthy	9	16.17	0.000	32.5	11.75	0.001
		25.3			53.3		
	Patient						
Overall prevalence of psychiatric disorders	Healthy	7.5	20.92	0.000	31.0	13.27	0.000
		23.1			49.6		
	Patient						

Data were analyzed using Chi-square test. p-value lower than 0.05 was considered to be significant.

In our study, 33% of PED users had experienced acute psychological disturbances after using these agents. The experience of acute psychological effect of PEDs was different in bodybuilders with psychiatric disorders compared to healthy athletes (χ^2 ^= 3.91, df = 1, p = 0.05). The prevalence of psychotic disturbances after using PEDs was significantly different in the athletes, who used various classes of PEDs (χ^2 ^= 13.24, df = 5, p = 0.02). Moreover, the highest level of psychotic disturbance after using PEDs was related to the athletes who had used 3 classes of these agents (AASs, stimulants, and peptides) (75%). Moreover, the prevalence of psychotic disturbances after using AASs was higher than the non-AAS users although the difference was not statistically significant (χ^2 ^= 4.17, df = 1, p = 0.06). Similarly, the prevalence of psychotic disturbances after using stimulant agents (33.9%) was not significantly different from non-stimulant users (32.2%) (χ^ 2 ^= 0.08, df = 1, p = 0.78). 

There were no significant differences between the current, non-current, and nonuser bodybuilders in the measured psychiatric disorders (Table 2). The type of PEDs abused by the athletes had no association with MDD (χ2(df) = 8.97(10), p = 0.56), schizophrenia (χ2(df) = 0.38(10), p = 1.00), and GAD (χ2(df) = 7.30(10), p = 2.00), and the overall prevalence of psychiatric disorders (χ2(df) = 8.03(10), p = 0.16). However, the type of PED class had a correlation with BD (χ2(df) = 31.64(10), p = 0.00). Those athletes, who used all classes of PEDs together, had a higher rate of BD (33.3%). Furthermore, anxiety was more prevalent only among stimulant agent users (25.5%), and mood disorders were more seen among athletes who had used 3 classes of PEDs (44.4%).

PED: performance-enhancing drugs, MDD: major depressive disorder, BD: bipolar disorder, GAD: generalized anxiety disorder, OPP: the overall prevalence of psychiatric disorders 

Family history and personal history of psychiatric disorders had no correlation with the initiation of PED use (rs = 0.07, p>0.05 and rs = 0.08, p>0.05, respectively). However, the length of PED use cycles (day/year) was associated with MDD (rs = 0.14, p = 0.03), GAD (rs = 0.20, p = 0.00) and the overall prevalence of psychiatric disorders (rs = 0.19, 

p = 0.00). Moreover, the FSH level was associated with MDD (rs = -0.15, p<0.05) and violence (rs = -0.17, p<0.05), while there was no association between FSH and other psychiatric disorders. 

Our study revealed that the prevalence of violence and alcohol use were higher in the current and non-current athletes compared with the nonuser athletes (χ^2 ^= 23.35, df = 2, p = 0.00 and χ^2 ^= 17.14, df = 2, p = 0.00, respectively) (Table4). Moreover, the prevalence of violence in athletes with MDD, BD, schizophrenia, and GAD was higher than the healthy athletes (Table 4). Moreover, the prevalence of alcohol use was higher in the athletes with MDD and GAD compared with the non-MDD and non-GAD athletes (Table 4). 

## Discussion

The present study revealed a high prevalence of psychiatric disorders among bodybuilders. There are limited research about the prevalence of psychiatric disorders in the athletes (15). However, it was shown that mental disorders are as common in sports as they are within the general population (15). Our study may imply that the prevalence of psychiatric disorders in bodybuilders was higher than the general Iranian population. When we comparing our study to a study about the prevalence of psychiatric disorders in the Iranian general population (16), we found that the overall prevalence of psychiatric disorders in the male bodybuilders may be higher than the general population. In line with our study, it was shown that the prevalence of mood disorders was higher in the bodybuilders compared with the non-athlete controls (17). We only considered male athletes, while other studies on the general population included both male and female participants. 

Our study demonstrated that the prevalence of psychiatric disorders was not significantly different between PED abuser (current and non-current) and non-abuser athletes. In this regard, there are some inconsistencies between the studies about the effects of PEDs, particularly AASs, on mental health (3, 18). In agreement with our study, it was found that PEDs, particularly AASs, exerted minimal long-term effects on the mood status (19). Furthermore, a study by Angoorani and Halabchi (20) revealed that mental health had no correlation with the AAS abuse in Iranian bodybuilders. On the other hand, some reports have shown that the prevalence of mood and anxiety disorders were higher in AASs abusers (5). In this regard, a Swedish research has shown that AASs ex-users were more engaged in seeking psychiatric treatment compared with the nonuser athletes (21). These inconsistencies may be related to the interindividual variation in both condition of androgen excess (22) and deprivation (23). Taken together, it is not completely clear whether AASs play a causative role in the development of long-term psychiatric disorders, but some evidences imply that AASs abusers may be at a greater risk for mood disorders (10). 

About one third of the bodybuilders in our study had experienced psychotic disturbances after using PEDs. Moreover, our study may imply that the prevalence of psychotic disturbances after using PEDs is higher in those athletes with psychiatric disorders. Furthermore, it found that using high doses of methyltestosterone raises the chance of acute mood changes in the male normal volunteers (11). Also, our study showed that using different classes of PEDs simultaneously may be another risk factor for the PED-induced acute psychotic disturbances. This may be related to synergistic effects of AASs and stimulants on the CNS. In this regard, it was shown that stimulant agents can produce psychoactive adverse effects such as anxiety, irritability, and paranoia (24). In addition, some studies have proposed that AAS abusers exhibit acute psychotic symptoms such as hypomania, irritability, and aggressiveness during exposure to these agents (25). Therefore, administration of these 2 classes of PEDs may increase the probability of acute psychiatric side effects of these agents. 

The length of PED use had a correlation with MDD and GAD in the bodybuilding athletes. Some studies have implied that mood changes regarding PED use may be related to deprivation from AASs (26). In contrast, our study may imply that PED use but not deprivation may be in association with the behavioral disturbances, mainly because increasing the length of PED cycles (day/year) use had increased the likelihood of psychiatric disorders.

The prevalence of aggressive behavior was higher in the PED abusers compared with the nonuser athletes. Moreover, our study revealed that the FSH level has a reverse correlation with aggressive behavior. This may imply that higher level of AASs may have a correlation with aggressive behavior. Also, in several other studies, it was revealed that AASs had profound effects on the aggression (27) and high dose of AASs elicit aggressive behavior in animals and humans (27, 28). However, the correlation of AAS use and aggressive behavior may be confounded by the psychiatric disorder in bodybuilders. Our study showed that the likelihood of aggressive behavior in the athletes with at least one psychiatric disorder was about 3 times than that of healthy athletes. Therefore, it seems that other studies that did not measure psychiatric disorders in the AAS abusers, might have overestimated the aggressive effects of AASs.

Our study showed that the likelihood of alcohol use was higher among PED users. AAS have been involved with alcohol and other illicit drugs (29). In contrast, some investigations did not report any association between alcohol and PED use (14). Similar to the aggressive effects, we reported higher rates of alcohol use among athletes with at least one psychiatric disorder. Again, our study may imply that the higher prevalence of alcohol use among PED users may be confounded by the presence of psychiatric disorders. Therefore, it is better to distinguish the effects of psychiatric disorders when comparing the correlation of AASs and alcohol administration.

## Limitation

We could not identify the current users of stimulants and peptide hormones. However, athletes almost always use other classes of PEDs together with AASs. Moreover, the classification of participants to non-current and nonusers mainly relied on the personal reports and some athletes might have hidden their status of PED use. Therefore, we suggest further studies on the psychiatric effects of PEDs in ex-users and users with established PED use background.

## Conclusion

Taken together, the prevalence of psychiatric disorders in bodybuilders is high and comparable (if not higher) with the general population. However, the prevalence of psychiatric disorders was not significantly different in PED user and nonuser bodybuilders. Therefore, it can be concluded that performance-enhancing drugs do not increase the risk of psychiatric disorders in bodybuilders.
